# Medical and Philosophical News

**Published:** 1782

**Authors:** 


### SECTION III.

### Medical and Philosophical News.

THE Royal Medical Society at Paris have propofed the following queftion for a prize of 400 livres: " What are the difeafes that " prevail the moft commonly among the troops " during the fummer, and in very hot weather; " what is the moft fimple and leaft expenfive " method of treating them, and laftly, what " are the means to be employed for preventing " or moderating their effects in very hot cli-" mates, as in the windward or leeward iflands?" The differtations on this fubject are to be written in Latin or French, and fent to M. Vicq D'Azyr, fecretary of the fociety, before the 1ft of December, 1783.

The Royal Academy of Sciences at Paris have offered a premium of 2400 livres to the person who shall: "find out the easiest and "cheapest process for decomposing sea salt, and "extracting the alkali, that serves as its basis, "in a state of purity, free from any acid or other "combination, provided the value of this mi-"neral alkali does not exceed the price of that "which is procured from the best foreign soda." The dissertations on this subject are to be written in French or Latin, and sent to the Marquis de Condorcet, secretary of the Academy, before Easter 1783.

The Academy have likewise proposed the following subjects for a prize of 1500 livres: "1. To make a chemical analysis of borax, of "sedative salt, and of the earth of crude borax "as it is brought from the East Indies; 2. To "make artificially, if it be possible, borax or "sedative salt, or some other saline substance "which may be employed as advantageously as "borax in the arts, and especially in the fusion of "metals; 3. To enquire whether there exists a "native sedative salt in any part of the world "except in the waters of the lake of Monte "Rotondo in Italy, in which it has already been "found."

The

The academy being ſenſible of the difficulties attending the ſatisfactory diſcuſſion of theſe three heads of enquiry, give notice, that if amongſt the diſſertations that may be ſent to them any one ſhall be found to contain new and important obſervations, the author's not having directed his reſearches to every part of the ſubject, ſhall be no obſtacle to his receiving the premium. The diſſertations are to be ſent to the ſecretary on or before the 1ſt of November 1783.

---

The Academy of Sciences at Toulouſe have propoſed the following ſubject for a prize of 100 piſtoles: "To aſcertain the effects which "air or aeriform fluids introduced into, or gene- "rated in the human body, have on the animal "œconomy." The diſſertations are to be written in French or Latin, and ſent to the ſecretary on or before the 31ſt of December, 1783.

---

No ſubject, perhaps, has engaged the attention of philoſophers of late years more than the queſtion concerning the nature of phlogiſton.

 By

By ſome it has been conſidered as the matter of fire; by others as the matter of light, or of both; while many, in conſequence of this uncertainty, have even gone ſo far as to deny its exiſtence. The ſagacity of Dr PRIESTLEY has at length enabled him to reſolve this difficult problem, as will appear by the following extract of a letter from that gentleman to one of his friends in London, dated Birmingham, March 12, 1782.

"I threw the focus of a burning lens on calx of lead in *inflammable air*; the air was rapidly abſorbed; lead was formed, and what remained of the air was as inflammable as ever.

"From forty ounce meaſures of the air, I revived about five pennyweights of lead. The inflammable air was from iron. In the ſame manner, I have revived iron, and tin. I alſo make ſtrong nitrous air by throwing nitrous vapour into inflammable air.

"This ſeems to prove that phlogiſton, is the very ſame thing with inflammable air in a ſtate of combination with bodies, exactly as fixed air is contained in chalk, &c. ſo that phlogiſton may be exhibited *by itſelf*, as well as any other ſubſtance in nature."

Dr.

Dr. Prieſtley has further obſerved, that in nitrous, and phlogiſticated air, only *glaſs of lead*, and not the *metal*, is obtained; and he is now buſied in proſecuting this important ſubject.

---

Extract of a letter from Sir T. Bergman, profeſſor of chemiſtry, at Upſal, &c. to a phyſician in London, (dated Upſal, Feb. 8. 1782.)

" Ab initio Januarii gravem hæmoptyſin ſuſtinui. Notatu eſt digniſſimum, quod aqua ſelterana, quâ antea per undecim annos molimina hæmorhoidalia, jam vehementiſſimam provocantia colicam, jam graviſſimos capitis dolores, ſedavit, excretiones quoque per pulmones efficacius, quam alia media, ad vias conſuetas, reduxerit. Scilicet intra quartam diem ſemper fluxum hæmorrhoidum provocavit et hoc ipſo mali cauſam aufert, nec non ſpaſmos temperat, ſanguinem ad ſuperiores partes urgentes. Ultimus impetus, primo autumnali longe vehementior, unicé per aquam ſelteranam ſublatus fuit et intra 8 dies eo uſque fui reſtitutus, ut melius valerem, quam per 3 menſes antea. Arte paratam unice bibo, ſed quin naturalis idem præſtet præſtet non dubito, et opera certe pretium erit in hæmorrhoidibus vel menſtruis ſuppreſſis idem tentare medium, quod ſaltim in perſonis meæ conſtitutionis ſalutarem præſtabit effectum.—Quod meæ diſſertationes non diſpliceant gaudeo. Eaſdem permagni ipſemet non debeo eſtimare, ſed vix in pueris quindecim annorum, ut tales conſcribant *, cadere poſſe confido. Ignorant, qui meam prolem eſſe dubitant, in quavis facultate bis eſſe diſputandum, priuſquam gradus conferatur apud nos. Prior diſputatio ſemper preſidem agnoſcit auctorem, et plerumque etiam poſterior. Si novas veritates probé intelligit et bene defendit Reſpondens egregium præſtaſſe ſpecimen putamus. Cæterùm titulum inſpiciant, et opuſcula ab auctore collecta reviſaque videbunt.—Morbus, quo laboravi impedivit, quo minus hoc anno prodire queat volumen tertium. Plures eo continebuntur, quæ memet in primis

* The learned profeſſor alludes here to a miſtake into which ſome perſons have fallen with regard to his eſſays, which from their having been originally publiſhed by ſtudents in the form of inaugural diſſertations have been erroneouſly conſidered as the productions of young and inexperienced writers. The reader may ſee one of thoſe diſſertations announced in our ſecond volume, page 141.

delectarunt delectarunt, ideoque poſtea variis novis experimentis eaſdem ditavi, ſuis locis inſerendis. Huc pertinent præcipue diſsertationes, *de phlogiſti copia in metallis*; *de ferri analyſi*; *de productis volcaneis*; *de attractionibus electivis*; et nonnulla alia."

---

A Literary and Philosophical Society hath lately been inſtituted at Mancheſter. The number of ordinary members is limited to fifty; but this limitation is not to preclude any number of gentlemen, diſtinguiſhed for their literary or philoſophical abilities, and reſiding at ſuch diſtances from Mancheſter as to prevent their attendance at the weekly meetings of the ſociety, from being elected honorary members. Mr. Henry, F. R. S. and Mr. George Bew are the ſecretaries of this new inſtitution.—The following amongſt other ingenious papers, have already been read before the ſociety:—1. Obſervations on Dr. Dobſons account of the Harmattan; by Alexander Eaſon, M. D. 2. Obſervations on heated rooms; by George Bell, M. D. 3. Miſcellaneous obſervations on the application of natural hiſtory to poetry; by T. Percival.

val. M. D. F. R. S. &c. 4. An account of ſome experiments made in a heated room; by Thomas Henry, F. R. S. 5. On the advantages ariſing from the inſtitution and well regulated ſupport of a philoſophical ſociety; by John Wright, M. D. 6. On the antiſeptic and chemical effects of quick lime on ſea water; by Mr. Henry. 7. On chryſtalliſation; by Dr. Eaſon. 8. A comparative view of the moral and intellectual powers of man; by Dr. Percival. 9. On the affinity ſubſiſting between the arts; by the Rev. Mr. Barnes. 10. On the different ſucceſs, with regard to health, of ſome attempts to paſs the winter in high Northern latitudes; by Mr. Aikin. 11. On the formation of ſalt-petre; by Mr. Maſſey.

---

Dr. Bloch of Berlin has obtained the prize of an hundred rix dollars, offered ſome time ago by the Royal Society of ſciences at Copenhagen, for the beſt diſſertation on the ſymptoms and treatment of worms in the inteſtines.

---

A caſe of Hydrophobia has occurred lately at the Middleſex Hoſpital. The patient, a middle aged aged man, was admitted into the hoſpital on the 15th of December 1781. He had been bit the ſame day, upon the cheek, by a large dog. The wound was of conſiderable extent, reaching from the lower eyelid to the left corner of the mouth. As there was no reaſon to ſuſpect that the dog was mad, common dreſſings were applied to the wound, and in leſs than three weeks after the accident it was perfectly healed, ſo that on the 8th of January 1782, the patient was diſcharged from the hoſpital apparently in good health. The next day he was brought back labouring under ſymptoms of Hydrophobia. The perſons who accompanied him to the hoſpital related, that he had that morning been thrown into convulſions by attempting to ſwallow a little tea; and that he ſeemed conſcious of his madneſs, having deſired his wife to take care that he did her no injury.

It was remarked that the part in which he had been bit looked a little red; this fact ſeems to confirm the truth of an opinion lately ſuggeſted relative to the hydrophobous infection; which is, that before the diſeaſe actually makes its appearance, a ſecond inflammation takes place in the wound or its cicatrix. That the patient had a very a very particular, uneaſy ſenſation in the part, is very evident from this circumſtance, that the moment he came into the hoſpital, he requeſted that ſomething might be applied to his cheek; and that from that time till his death he was obſerved, when free from convulſions, to be almoſt inceſſantly putting his hand to his tongue and carrying it from thence to the cheek in which he had been bit.

When he was re-admitted into the hoſpital he appeared to be much agitated; his tongue was white, and his pulſe full and conſiderably quickened. The ſight of water did not then or at any time afterwards give him the leaſt uneaſineſs, but whenever he attempted to drink he was immediately ſeized with ſpaſms of the throat and with violent effort to vomit.

To combat this melancholy diſeaſe every method was adapted that the reading and experience of the learned and ingenious phyſician who had the care of the patient, could ſuggeſt to him. Mercurial ointment was applied to the cicatrix in the cheek; and muſk, cinnabar, and a variety of other remedies were preſcribed internally but without the deſired effect.

When

When the patient tried to ſwallow his medicines he took them haſtily from the point of a knife, but though he chewed and was very deſirous to ſwallow them, he ſeldom ſucceeded, getting down only a very ſmall portion of them and that with difficulty. During the whole of the firſt day (Wedneſday January the 9th) he gave rational anſwers and readily attempted to do whatever was deſired. He paſſed the night without the leaſt diſpoſition to ſleep. His pulſe became ſmaller and quicker, and he was troubled with a conſtant ſlavering of a thick frothy mucus. The next day (January the 10th) about one o'clock in the afternoon his difficulty of ſwallowing ſeemed to be leſſened, as he was able to get down a little water out of a tea-pot, but ſoon after this he became outrageous and was obliged to be bound to his bed. In this ſtate he continued till his death which happened about three o'clock.

Upon diſſection after death, the ſurface of the tongue and fauces was found to be white but not dry. The muſcles of the tongue were apparently ſound; the ſubmaxillary glands ſomewhat enlarged; the œſophagus ſlightly inflamed, and the lymphatic glands near it larger than common.

The

The ſtomach and duodenum contained a large quantity of yellowiſh bile. The reſt of the abdominal viſcera afforded no remarkable appearance. The lungs ſeemed to be rather fuller of blood than uſual, and adhered at different parts to the pleura. The veſſels and ventricles of the heart were quite empty, and the *liquor pericardii* was in very ſmall quantity. On examining the brain the dura mater appeared to be in a contracted ſtate, and near the longitudinal ſinus it adhered firmly to the pia mater. A ſmall quantity of a watery fluid was obſerved between theſe two membranes at the poſterio part of the brain. The ſinuſes appeared to be more turgid than uſual, but the quantity of water in the ventricles did not ſeem to be greater than what is natural.

---

Some experiments made by Mr. Mönch, an ingenious German apothecary, and publiſhed in the firſt part of Profeſſor Gells *Neueſte Ent dukungen in der chemie* for 1781, prove that calcined magneſia is leſs ſoluble in acids than hath been generally imagined. Theſe experiments were made with magneſia precipitated from Epſome fom falt, and calcined in a covered crucible after having been edulcorated with repeated wafhings with diftilled water.

Upon twenty grains of this calcined magnefia, in a clean glafs veffel, Mr. Mönch poured half an ounce of ftrong vinegar. No effervefcence enfued, and after fuffering the mixture to ftand for twenty four hours only feven grains of the magnefia were found to be diffolved. The fame experiment was afterwards repeated with frefh lemon juice inftead of vinegar, and with the fame refult, excepting that inftead of feven, the lemon juice diffolved only four grains of the twenty. Mr. Mönch next poured an ounce of vinegar upon twenty grains of the fame calcined magnefia; the mixture was boiled for a confiderable time, but only ten grains of the magnefia were diffolved.

Mr. Mönch took half an ounce of a mixture compofed of ftrong vitriolic acid one part, and water fix parts. This was poured on twenty grains of the calcined magnefia, and placed in a moderate degree of heat; but at the end of twenty hours only feven grains of the magnefia were diffolved. An ounce of the fame diluted acid of vitriol boiled for a conſiderable time with the ſame quantity of magneſia as in the former experiment, diſſolved only nine grains of it.

The nitrous acid and concentrated marine acid were found to diſſolve the magneſia entirely, but no ſuch effect was produced by mixing it with the gaſtric juice of different animals. Even the gaſtric juice which was vomited up by an hypochondriacal patient, altho' it effervesced ſtrongly with powdered crabs-eyes, diſſolved only two grains out of ten of the calcined magneſia.

---

Two new medical works are expected ſoon to be publiſhed in London: one of them, entitled "*Fragmenta Medica et Chirurgica*," is the production of Dr. William Fordyce; the other is a practical tract on the Venereal diſeaſe, the author of which is Dr. F. Swediar.—At Edinburgh, Dr. Cullen is ſaid to be preparing for the preſs a treatiſe on Nervous diſeaſes.

