# Errata

**Published:** 1782

**Authors:** 


					p. 94., /. 17, for
eftort r? /</ettorrs $ T. 19, for adapted read adopted 5 p. 96, /. i\> Jo*
poH:eno~n?<7</ pofterior ; A 20i for Ce-ll's read Call's j /. 27, for Epfome
read hipfom ;

				

